# Supplementary material for: Chloride-induced corrosion of steel in concrete—insights from bimodal neutron and X-ray microtomography combined with ex-situ microscopy
Source: Mater Struct. 2024 Apr 8;57(4):56. doi: 10.1617/s11527-024-02337-7 (PMC11001691; doi:10.1617/s11527-024-02337-7)
Supplement: Supplementary file 1 — Supplementary file1 (PDF 1157 kb) [file 11527_2024_2337_MOESM1_ESM.pdf]

Supplementary Information (SI)
for manuscript

Chloride-induced corrosion of steel in concrete – insights from bimodal neutron and X-ray microtomography combined with ex-situ microscopy

Ueli M. Angst [a]*, Emanuele Rossi [a], Carolina Boschmann Käthler [a,b], David Mannes [c], Pavel Trtik [c], Bernhard Elsener [a], Zhou Zhou [d], Markus Strobl[c,e]

[a] Institute for Building Materials, ETH Zurich, Zurich, Switzerland
[b] Hagerbach Test Gallery Ltd., VSH, Flums, Switzerland
[c] Laboratory for Neutron Scattering and Imaging (LNS), Paul Scherrer Institut, Villigen, Switzerland
[d] Department NPM2/RST, Faculty of Applied Sciences, Delft University of Technology, Delft, The Netherlands
[e] Niels Bohr Institute, University of Copenhagen, Copenhagen, Denmark

* corresponding author: uangst@ethz.ch

A. Supplementary Information

A.1. Potential measurements of reinforced concrete specimens

The potential measurements to electrochemically monitor the corrosion onset of reinforced concrete cores are reported in Figure S1.

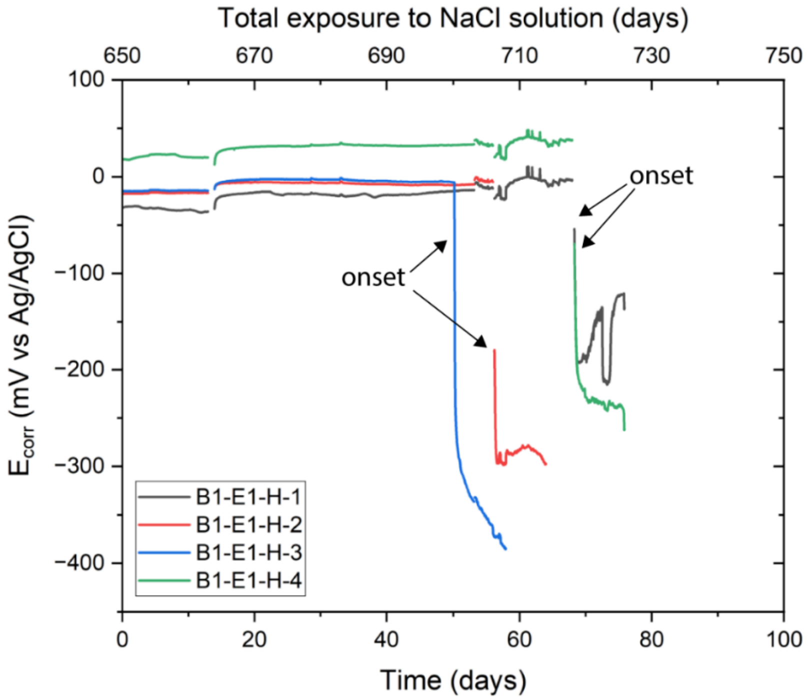

Figure S1. Potential measurements of reinforced concrete specimens over time (vs. Ag/AgCl/KCl_{sat}), after which coring and X/n computed tomography scanning was conducted. Only the last measurements before corrosion onset are reported (the total duration of the exposure to NaCl solution is visible from the top x-axis)

A.2. Comparison between segmentation procedures based on singular or bivariate histograms

An example of the results of the segmentation procedure based on the images' gray-scale value (GSV) and bivariate histograms is visible in Figure S2-S3. For this example, a 2D cross-section image of the reinforced concrete specimen has been considered. Segmentation of both X-rays and neutron scans of the same cross sections has been performed by thresholding the respective GSV histograms (Figure S2), and the segmentation results are visible in Figure S3. Segmentation has been also performed using the bivariate histogram of the same cross-section (previously reported in the core manuscript, Figure 5). A K-Means clustering algorithm was used to differentiate the specimen's components as a basis for further segmentation. The pixel clusters related to aggregates, cement paste, voids, and background could be identified. On the other hand, the beam hardening effect present at the steel-concrete interface (SCI) made the segmentation between steel and corrosion products less sharp. To overtake this limitation, the steel region of the image was segmented via the X-ray GSV histogram, while the corrosion products were segmented via the neutron GSV histogram. The absence of any beam hardening effects would potentially make this procedure completely automatic. Since in the present paper no quantitative analysis was performed, the final segmentation allowed to gather the necessary information for the purpose of the study (e.g., measuring the corrosion attacks' dimensions, observing the interfacial characteristics where they occurred, counting and measuring the dimensions of interfacial air voids, etc.). Nevertheless, the removal

or reduction of beam hardening effects is crucial for further studies that would focus on the micro-scale details of the SCI as well as on quantitative analysis of high number of images. The results of the segmentation based on the bivariate histogram of the present 2D cross-section are visible in Figure S3.

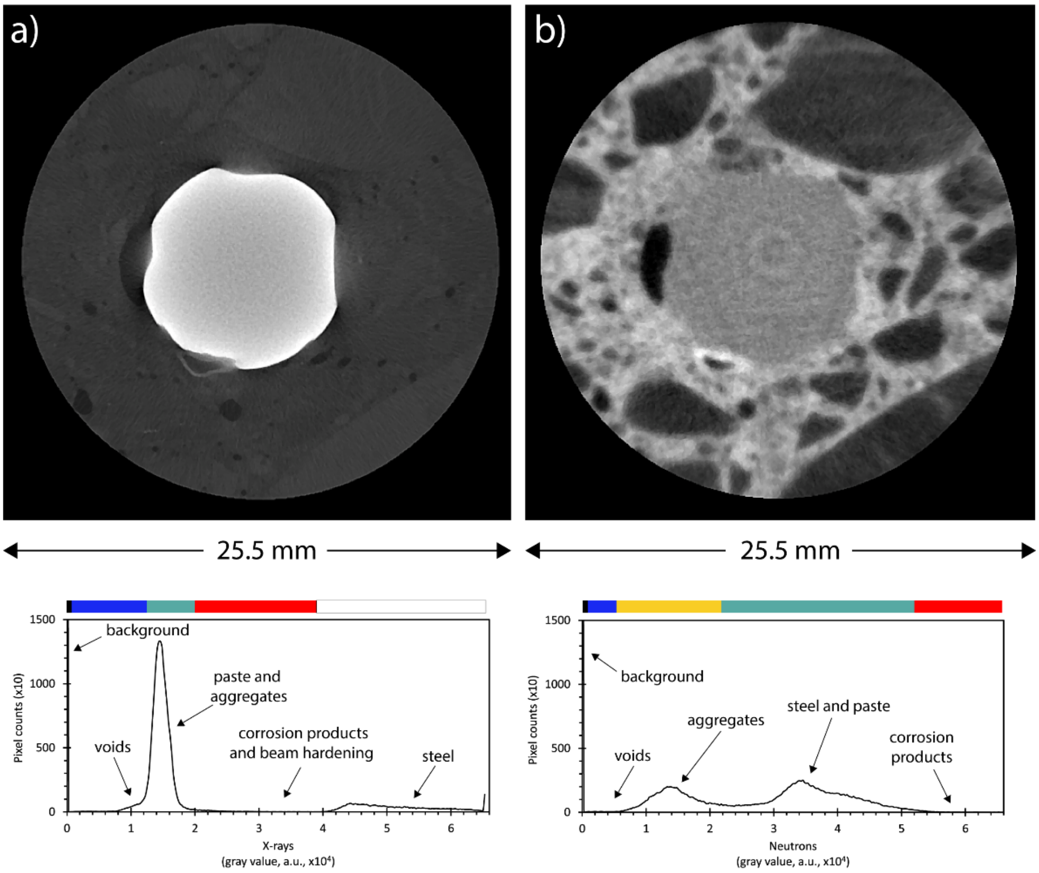

Figure S2. 2D X-ray (a) and neutron (b) cross-section images, with respective grayscale value (GSV) histograms and color ranges of the different segmented components.

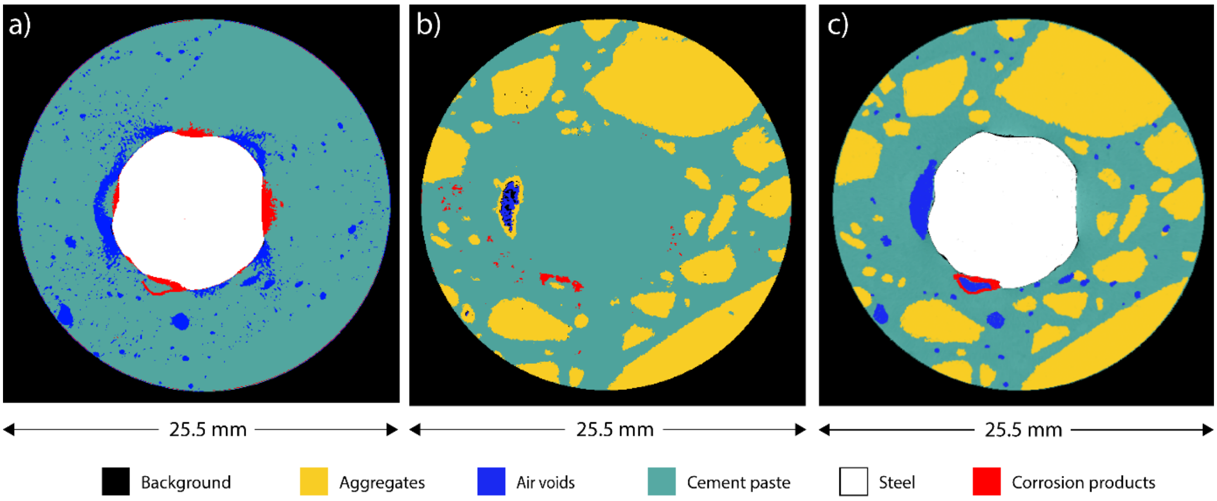

Figure S3. Results of the segmentation procedure using the X-rays GSV histogram (a), the neutron SGV histogram (b), and the respective bivariate histogram (c).
